# Telomere Length in Human Adults and High Level Natural Background Radiation

**DOI:** 10.1371/journal.pone.0008440

**Published:** 2009-12-23

**Authors:** Birajalaxmi Das, Divyalakshmi Saini, M. Seshadri

**Affiliations:** Radiation Biology and Health Sciences Division, Bhabha Atomic Research Centre, Trombay, Mumbai, India; National Cancer Institute, United States of America

## Abstract

**Background:**

Telomere length is considered as a biomarker of aging, stress, cancer. It has been associated with many chronic diseases such as hypertension and diabetes. Although, telomere shortening due to ionizing radiation has been reported *in vitro*, no *in vivo* data is available on natural background radiation and its effect on telomere length.

**Methodology/Principal Findings:**

The present investigation is an attempt to determine the telomere length among human adults residing in high level natural radiation areas (HLNRA) and the adjacent normal level radiation areas (NLNRA) of Kerala coast in Southwest India. Genomic DNA was isolated from the peripheral blood mononuclear cells of 310 individuals (HLNRA: N = 233 and NLNRA: N = 77). Telomere length was determined using real time q-PCR. Both telomere (T) and single copy gene (S) specific primers were used to calculate the relative T/S and expressed as the relative telomere length. The telomere length was determined to be 1.22±0.15, 1.12±0.15, 1.08±0.08, 1.12±0.11, respectively, among the four dose groups (≤1.50, 1.51–3.00, 3.01–5.00 and >5.00 mGy per year), which did not show any dose response. The results suggested that the high level natural chronic radiation did not have significant effect on telomere length among young adult population living in HLNRA, which is indicative of better repair of telomeric ends. No significant difference in telomere length was observed between male and female individuals. In the present investigation, although the determination of telomere length was studied among the adults with an age group between 18 to 40 years (mean maternal age: 26.10±4.49), a negative correlation was observed with respect to age. However, inter-individual variation was (0.81–1.68) was clearly observed.

**Conclusions/Significance:**

In this preliminary investigation, we conclude that elevated level of natural background radiation has no significant effect on telomere length among the adult population residing in HLNRAs of Kerala coast. To our knowledge, this is the first report from HLNRAs of the world where telomere length was determined on human adults. However, more samples from each background dose group and samples from older population need to be studied to derive firm conclusions.

## Introduction

High Level Natural Radiation Areas (HLNRA) in the world provide ample opportunities to study the biological and health effects of natural chronic low level radiation directly on humans. The level of natural background radiation in these areas is sometimes ∼10–100 times higher than normal areas. The level of background radiation is high either due to hot springs with high radium contents like Ramsar in Iran or due to monazite bearing sand in Yangjiang in China, Guarapari in Brazil and and the coastal belt of Kerala in south India. The monazite bearing sand contains thorium and its daughter products. The non-uniform distribution of radiation exposure prevailing in monazite bearing HLNRA of Kerala coast in South west India (contains 8–10% of thorium, highest in the world) gives an unique opportunity to conduct *in vivo* dose response studies on humans. This costal belt is approximately 55 kms long and 0.5 km wide, extending from Neendakara (Kollam district) in south to Purakkadu (Alapuzha district) in north [1]. It is thickly populated and the total population in this area is about 4,00,000 with approximately one third residing in high level natural radiation area and the rest in adjacent control area (normal level natural radiation area). The human population residing there is more than 50 generations old. Thus, it is considered as an ideal place to study the long term detrimental effects if any due to chronic natural low dose radiation on human population.

It has been estimated that the per capita average dose received by the population residing in HLNRA is ∼4 mGy/per year [2], [3]. However, the background level in this area ranged from ≤1.0 mGy to over 45 mGy per year [4]. For the past few decades, human population living in HLNRA of Kerala coast is under investigation to study the genetic effects of radiation on newborns and adult population. The background radiation dose ≤1.5 mGy per year is considered as normal level natural radiation area (NLNRA), whereas the background radiation dose above 1.5 mGy per year is considered as HLNRA. The percentage of samples available from the highest level of natural radiation area (>5 mGy per year) accounts for only 8–10% of the population.

The average annual effective dose from natural background radiation in the world is about 2.4 mSv [5]. At the age of 65 years one can accumulate about 160 mSv [6]. It is essential to understand whether the dose received in the areas of high level natural background radiation can induce detectable level of biological effects using the established cytogenetic parameters like dicentrics, micronuclei etc. It is possible to detect the effect of an elevated dose level of natural radiation using a reasonably large number of cells per individual and analyzing many individuals [7]. Even assuming a mean external effective dose of 2.0–6.0 mSv per year as might have received in high background radiation areas like China, Iran, India and certain areas of Brazil, one can study the biological and health effects in the population [8].

Since decades, a comprehensive programme in collaboration with Bhabha Atomic Research Centre and Kerala State government is investigating the possible biological and health effects of high level natural background radiation on human population. Several investigations conducted in this area revealed that back ground radiation dose has no detrimental effects on the human population residing in this area. For instance, the cytogenetic investigation based on over 10,000 newborns did not reveal any significant difference in the frequency of chromosomal aberrations (both stable and unstable) [4], [9]. Screening of over 100,000 newborns for congenital malformations did not reveal any significant increase in the frequencies of malformations in HLNRA as compared to NLNRA [4], [10]. Cytochalasin blocked micronuclei assay on newborns also did not reveal statistically significant increase in the frequency of micronuclei (MN) among the newborns studied from HLNRA as compared to NLNRA [11].

Genotoxic agents and stress including ionizing radiation is known to cause significant DNA damage and chromosomal/chromatid aberrations. Telomeres are specialized nucleo-protein complexes that serve as protective caps of linear eukaryotic chromosomes. It consists of tandem repeats of the DNA sequence (TTAGGG)n at the end of the chromosomes. It not only plays an important role in maintaining chromosome stability but also helps in protecting of the coding parts of the DNA for recombination, degradation and replication damage. Telomere shortening is a biomarker of cellular senescence and is known to be associated with age related disease [12]. In recent years, the telomere length determination is increasingly being used to study the effect of aging and stress, although it is not yet established as a biomarker [13], [14]. Many studies have shown that average telomere length in white blood cells shortens with ageing, although individual variation within the same age group exists. Telomere length shortening is also associated with oxidative stress [12], [15], [16].

Shortening of telomere length has been reported to be associated with aging, stress, diabetes, hypertension, many other age related diseases and cancer. Few reports are available on telomere length variation in response to ionizing radiation. However, no *in vivo* data on telomere length in response to elevated level of back ground radiation is available. Several experimental methods have been developed to determine telomere length including southern blot, fluorescence in situ hybridization (Q-FISH) analysis [17], Flow-FISH analysis [18] and most recently, by quantitative PCR [19]. In the present investigation, we have made an attempt to measure the telomere length in random, normal, healthy, age matched young adults (18 to 40 years) from high level natural radiation areas (HLNRA) and adjoining normal level radiation areas (NLNRA) of Kerala coast to assess the effect of elevated level of natural background radiation, if any, on telomere length. For that purpose, we have used an established method developed by Cawthon et al in 2002 [19], where, telomere length was determined by using real time q-PCR. This method provides relative results about telomere length by calculating the ratio of a PCR reaction product from the same sample using specific primers for telomeres and single copy gene (T/S ratio).

## Results

### Relative Telomere Length

**Figure 1:**
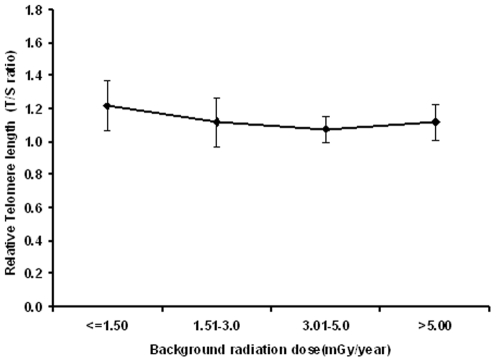
Telomere length in adults with respect to various background dose levels. (The line graph represents the relative telomere length (T/S ratio) in five different background dose groups (≤1.5, 1.51–3.0, 3.01–5.0, >5.00 mGy/year). For each point, error bars indicate the standard error of the mean (SEM). N (Number of individuals studied in each dose group) is given in table 1).

In the present study, telomere length was determined from the peripheral blood mononuclear cells (PBMC) of a total of 310 normal and healthy young adults (141 males and 169 females) from high and normal level natural background radiation areas of Kerala coast in south west India. The study samples included 233 adults from HLNRA and 77 adults from NLNRA with mean maternal age of 26.24±4.52 years and 25.69±4.38 years, respectively. Inter-individual differences in telomere length were clearly observed among these individuals studied. In order to assess if there is any dose dependant relationship between telomere length and background radiation dose, we have divided the study samples into four groups based on background radiation dose levels i.e., ≤1.50 mGy per year, 1.51–3.00 mGy per Year, 3.01–5.00 mGy per Year and >5.00 mGy per year (as given in table 1, figure 1). The samples belonging to >5.01 mGy per year was not grouped further due to the limitation of samples size in that particular dose group.

**Table 1:** Telomere length in various background dose levels and 95% Confidence Intervals (CI).

Area	Background Radiation dose range in mGy/Year (mean)	No. of samples analysed	Telomere length (T/S ratio)±SD	95% CI	
				Lower	Upper
NLNRA	≤1.50 (1.10)	77	1.22±0.15	1.1840	1.2532
HLNRA	1.51–3.00 (2.00)	147	1.12±0.15	1.0937	1.1419
	3.01–5.00 (3.79)	50	1.08±0.08	1.0541	1.0977
	>5.00 (18.67)	36	1.12±0.11	1.0777	1.1545
NLNRA+HLNRA	Grand total (4.00)	310	1.16±0.14	1.1197	1.1511

NLNRA = Normal Level Natural Radiation Area, HLNRA = High Level Natural Radiation Area, T/S ratio = Telomere Specific gene/Single copy gene, SD = Standard Deviation.

**Figure 2:**
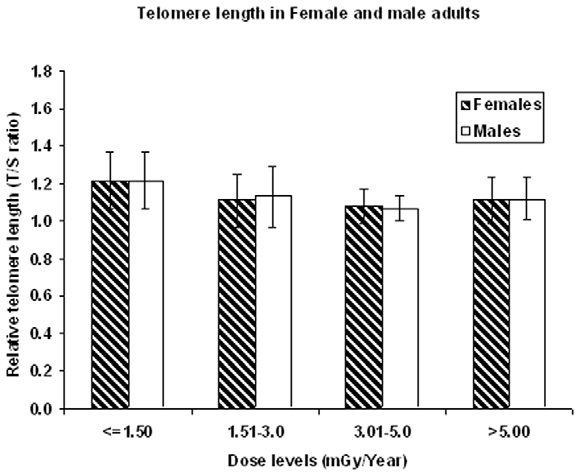
Telomere length among male and female adults with respect to various background dose levels. [(≤1.50 mGy/year: 34 males+43 females, 1.51–3.0 mGy/year: 67 males+80 females, 3.01–5.0 mGy/year: 23 males+27 females, >5.00 mGy/year: 17 males +19 females)].

We have analysed the telomere length data with respect to male and female adults in order to assess the gender difference if any. Our data revealed that the mean telomere length was 1.14±0.04 (mean age: 28.98±4.01 years) among males and 1.13±0.03 (mean age: 23.70±3.30 years) in females with an overall mean of 1.14±0.14 among all the individuals studied (mean age: 26.10±4.49 (range: 18 to 40 years). The values obtained in males was not statistically different from females (P>0.05) (figure 2). No dose response was observed among male and female adults with respect to all the four dose groups analysed.

**Figure 3:**
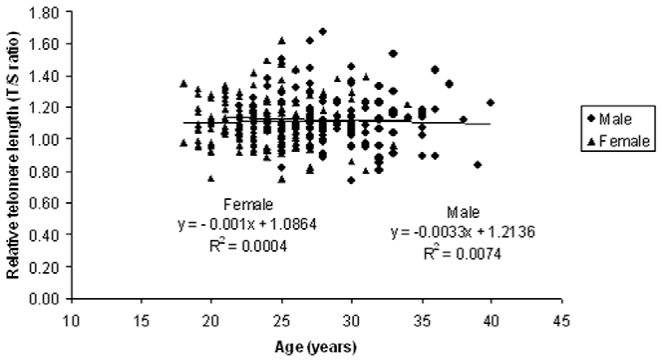
Telomere length in male and female adults with respect to age.

We have also performed regression analysis to study the relationship between age and telomere length. Taking all the samples into consideration, our results showed a significant decrease in the mean telomere length (R^2^ = 0.00002, y = −0.0002x +1.1147) with respect to age (figure 3). Similar trend was also observed when regression analysis of the telomere length with respect to age was performed among male (R^2^ = 0.0004, y = −0.001x +1.0864) and female (R^2^ = 0.0074, y = −0.0033x +1.2136) individuals separately.

Analysis of covariance (ANCOVA) was performed in order to adjust the influence of age and gender, if any, on telomere length. After adjusting the effect of age and gender, the mean telomere length was determined. In table 1, mean telomere length in all the four dose groups and the 95% confidence Interval (CI) was given. The mean telomere length was observed to be 1.22±0.15 (95% CI, 1.18–1.25) in NLNRA, 1.12±0.15 (95% CI, 1.09–1.14), 1.08±0.08 (95% CI, 1.05–1.09), 1.12±0.11 (95% CI, 1.07–1.15) in HLNRA dose groups. By taking gender alone as a variable the mean telomere length remained unchanged. The line graph in figure 1 showed the telomere length with respect to all the four dose groups. It did not indicate any dose related reduction in telomere length among these four dose groups. Moreover, the telomere length is almost similar among all the three stratified HLNRA groups. Interestingly, the mean telomere length was found to be >1.00 in all the four dose groups studied. As shown in table 1, the telomere length in various dose groups in HLNRA were not statistically different as compared to NLNRA (P>0.05).

Analysis was also done on the basis of shorter and longer telomere length. We have compared the individuals from each group with shorter telomere length (defined as relative T/S ratio below the median) versus longer telomere length (defined as relative T/S ratio above the median). Logistic regression was used to compute the odds ratios (OR) and 95% confidence intervals (CI), for individuals with shorter and longer telomere lengths in all the three HLNRA dose groups (>1.5 mGy per year) with respect to the NLNRA (dose group ≤1.5 mGy per year), where odds ratio was taken as 1.00. As shown in table 2, the OR values found in various dose groups in HLNRA were not statistically different as compared to NLNRA.

**Table 2:** Odds ratio and CIs of HLNRA and NLNRA adults according to telomere length.

	Odds ratio (95% confidence interval for dose group of 1.51–3.00 mGy/year)	Odds ratio (95% confidence interval for dose group of 3.01–5.00 mGy/year)	Odds ratio (95% confidence interval for dose group of >5.00 mGy/year)
Shorter telomere length vs longer telomere length	0.846 (0.486–1.473)	0.928 (0.454–1.898)	0.988 (0.446–2.192)

## Discussion

Regulation of telomere length plays an important role in the maintenance of genome stability in human. Although, research has been progressed in understanding telomere biology at molecular and cellular level, very little is known about the causes of telomere attrition. In recent years, there are innumerable reports showing telomere attrition due to infections and inflammation [20], exposure to infectious agents and other types of oxidative stress [15], [21], cardiovascular diseases [22]–[24], hypertension [25], [26], [27], diabetes [28], [29], dementia [30]. All these may cause damage to telomeric ends and impair their repair mechanisms [31]. Telomere shortening has also been reported in many cancer patients due to rapid cell proliferation [32]. Telomere shortening may play a vital role in biological aging [13]. There are many reports showing that oxidative stress induces increased erosion at telomeric ends resulting telomere length shortening [12], [15], [16], [33].

Telomeres play an important role in the formation of chromosome and chromatid aberrations induced by ionizing radiation [34]. Many cytogenetic studies have shown association of telomeric end fusion in metaphase spreads exposed to ionizing radiation. *In vitro* studies have revealed a difference in average telomere length after X-irradiation using quantitative analysis of emitted fluorescence (Q-FISH) [35], [36]. Studies on short and long term cultures have shown elongation of telomere length after exposure to low and high LET radiation suggesting better repair of telomeric ends [37]. *In vitro* experiment has also shown that telomere stability is correlated with longevity of humans exposed to ionizing radiation [38]. However, no *in vivo* data is available regarding telomere length in humans exposed to chronic low dose ionizing radiation. The high level natural radiation areas in the world provide such opportunities to study the effect of natural chronic low dose radiation. In the present investigation, we have determined the telomere length among younger human adults from Kerala coast in South India, where the human population is exposed to chronic low dose exposures to background radiation (∼50 generations). Confounding factors such as age, gender and other habits etc. are part of the population studies. Therefore, to delineate the effect of ionizing radiation, if any, on the telomere length of the studied individuals, it is necessary to do the dose response study. In general, a linear relationship would give an indication of radiation induced effects, if any. To find out the effect of natural background radiation on telomere length, our efforts are therefore to do the dose response analysis taking the control group (NLNRA: ≤1.50 mGy/per year) in addition to three HLNRA dose groups with different background dose levels ranging from ≥1.5 mGy per year to 28 mGy/year. As shown in figure 1, with the increasing background dose levels (≤1.50 mGy per year, 1.51–3.00 mGy per Year, 3.01–5.00 mGy per Year and >5.01 mGy per year), we did not observe dose related reduction of telomere length. The telomere length was almost stabilized in later dose groups from high level natural radiation areas. This finding suggests that the natural chronic low background radiation dose did not have significant influence on telomere length. Assuming that the high level of background back ground radiation can induce increasing DNA damage with increasing background dose level, we could have expected significantly shorter telomeres in the higher dose groups and in a dose dependant manner. On the contrary, we have observed similar telomere length in all the three HLNRA dose groups (1.12±0.15, 1.08±0.08 and 1.12±0.11) which. was not statistically significant (P>0.05). No clear-cut decrease in telomere length was observed, suggesting a better repair of telomeric ends in natural background area.

Telomere attrition is known to be correlated with DNA damage response [31] leading to detection of significant DNA damage. Micronuclei formation is one of the indicator of radiation induced DNA damage. This area is under investigation since many years. For micronuclei study, 271 newborns were analysed in peripheral blood lymphocytes in this study area (61 samples from NLNRA and 210 from HLNRA) using cytochalasin blocked micronuclei assay. Over one million binucleated cells were scored. This study did not report increased micronuclei frequency in HLNRA as compared to NLNRA [11]. Similarly, dicentric formation is another indicator of radiation induced DNA double strand breaks. Between 1986 and 2000, chromosomal aberrations were analysed in the peripheral blood lymphocytes (PBLs) of 14,217 newborns in the Kerala coastal belt whose mothers were exposed 1.5 mGy per year or more and in the PBLs of 5719 newborns whose mothers were exposed to less than 1.5 mGy per year [9], [39]. The cytogenetic investigation included both stable and unstable aberrations. A total of nearly 1 million metaphases were scored. The frequency of dicentric did not increase in HLNRA population as compared to NLNRA [9]. No correlation was found between the background radiation dose and the frequency of chromosomal abnormalities. The data also did not reveal any significant difference in the frequency of other types of chromosomal aberrations such as translocations, inversions, fragments/minutes, chromosome and chromatid breaks/gaps etc., [9]. All these data are supportive of a better repair system/adaptive response in the human population residing in that area. DNA repair study and transcriptome analysis on a statistically significant number of individuals are in progress in order to address some of these issues (unpublished data).

There are reports, which suggest that telomere length in germinal cells is maintained where as, in somatic cells it decreases with age [40], [41]. In most of the somatic cells, telomerase activity is usually not found [42], [43] except few observations [44], [45]. The real time q-PCR methodology used in our study is an established method by Cawthon [19], [46] for measuring relative telomere length which is correlated with telomere length obtained from Southern blot analysis by many investigators. Recently there are studies, which have shown that the telomere length can be calculated in terms of base pairs from the relative telomere length [46]. It is to be pointed out here that the telomere length detection by southern blotting covers the sub-telomeric region whereas the primers designed by Cawthon in 2002 [19] detect only the telomere specific repeat. Preliminary data from our laboratory on transcriptome analysis using affymetrics gene expression arrays (human genome U133 plus 2.0) on adults from HLNRA and NLNRA did not reveal increased level of expression of genes, which are associated with telomere length and its stability (unpublished data). For instance, the mean level of expression at telomerase reverse transcriptase (TERT) did not show any increase in expression in HLNRA adults as compared to control. The level of expression at other telomere associated proteins such as TEP1 (telomerase-associated protein 1), tankyrase, the TRF1-interacting ankyrin-related ADP-ribose polymerase (TNKS), Tankyrase, TRF1-interacting ankyrin-related ADP-ribose polymerase 2 (TNKS2) and TERF1 (TRF1)-interacting nuclear factor 2 (TINF2) are similar in both HLNRA and NLNRA (control) samples. All of these genes have shown a fold change of less than 1.3 (P>0.05) (Unpublished data). This supports our present data obtained from relative telomere length in HLNRA and Control adults. It also rules out methodological errors, if any. Measurement of telomere length by using real time PCR is an accepted method of choice from human blood samples as it requires small quantity of DNA. That is the reason this method may be useful for forensic invesigations [47], [48].

Telomere length and its association with aging have significant implications to human health [20]. Telomere length has been negatively correlated with aging and age related diseases [49], [50], [51]. In the present study, although our main objective was to study whether there is any difference in the telomere length due to the effect of chronic natural background radiation, we also looked at the telomere length with respect to age. Several studies have reported that telomere length becomes shorter with the advancement of the age [47], [48], [50]. In other words, shorter telomere length is observed in older age groups as compared to younger age groups. In spite of analyzing samples from a narrow age group, we have observed a negative correlation between the telomere length and the age (R^2^ = 0.00002, y = −0.0002x +1.1147, P<0.001). Similar trend was found both in male and female individuals.

We have analysed our data to find out the difference of telomere length with respect to both the sex. There are many studies showing significant difference in telomere length among male and female adults. Many studies have shown that the telomere length in females is significantly longer than males [13], [32], [47], [50]. In the present study, we did not observe any significant differences in telomere length in male and female adults with respect to all the above five background dose groups as shown in figure 2. This finding is also similar to our cytogenetic findings in male and females newborns and the micronuclei frequency.

The interesting finding in this preliminary study is the average telomere length (T/S) in HLNRA and NLNRA is 1.0 in majority (approximately 96%) of the individuals studied, which clearly indicates that elevated level of natural background radiation has no significant effect on telomere length in high background radiation areas of Kerala coast. The limitation of our study is that we need to collect more individuals from the older age group. Perhaps cumulative radiation dose might give a clear picture of telomere length among the adults from all the age groups. Since the population is living in this area for at least 50 generations, the adult samples analysed in the present study have sufficient exposure to low dose natural radiation during their life. Although our limitation is we have studied adults of a younger age group, older population needs to be anlysed to have a clear-cut picture of the cumulative exposure effect in their life time. To our knowledge, this is the first *in vivo* study which attempts to address the dose response relationship between natural chronic background radiation and telomere length in human peripheral blood mononuclear cells.

The presence of large inter-individual variation in telomere length, telomere length attrition between males and females, telomere length loss during different stages of life, association of telomere length and age, genetic factors and environmental factors give rise to the large amount of variation of telomere lengths a population. Therefore, studies pertaining to telomere length regulation during the life span might provide a clearer picture of the association between telomere length and age.

## Materials and Methods

### Subjects

In the present investigation, venous blood samples were obtained from 310 normal, healthy adults (141 males and 169 females) with a mean maternal age of 26.34±3.81 (age range: 18–40 years). The samples were collected through four hospital units located in HLNRA and the adjoining NLNRA (control areas) of Kerala coast in south India. The samples from HLNRA included 107 males and 126 females (mean maternal age: 26.24±4.52), whereas 34 males and 43 females (mean maternal age: 25.69±4.38 years) were from NLNRA. The study was approved by the institutional ethic committee of Bhabha Atomic Research Centre, Mumbai and all the participants gave written informed consent. Data pertaining to life style, occupation, duration of stay in HLNRA, habits etc. are recorded in proformae designed under the World Health Organization (WHO) guidelines. The average radiation levels for the family are incorporated for each adult individual and the data entry is verified and validated for internal consistency. The life style and diet of the adult individuals was similar in both HLNRA and NLNRA, because of the similarities in the socio-economic status.

The study area is a coastal belt of about 55 km long and 0.5 km wide and the radiation dose ranges from 1.0 mGy per year to 45 mGy per year. The average radiation dose in NLNRA of Kollam district is 1.2 mGy per year with a range of <1.0 to 1.5 mGy per year. Hence area with an exposure above 1.5 mGy per year is considered as High Level Natural Radiation area (HLNRA). The classification of high level and Normal level natural radiation is based on the level prevailing at the residence of the individual. Greiger Muller Counters were used for measuring the terrestrial gamma radiation and average area dose was calculated from 0.5 Km grid.

### Telomere Length Measurement with Real Time PCR

Genomic DNA was extracted from peripheral blood mononuclear cells (PBMC) using a rapid non-enzymatic method [52]. Relative telomere length was determined by using the approach as previously described by Cawthon in 2002 [19] with a little modification in the PCR temperature conditions. Relative telomere length was determined from the genomic DNA obtained a total of 310 individuals ( 141 males and 169 females) from HLNRA and NLNRA of Kerala coast by quantitative real time polymerase chain reaction (PCR). This method measures the factor by which the ratio of telomere repeat copy number to single – gene copy number differs between a sample and that of a reference DNA sample. PCR amplification was achieved using telomere (T) and single copy gene, 36B4 (encodes acidic ribosomal phosphoprotein) primers(S) which serves as a quantitative control. The mean telomere repeat gene sequence (T) to a reference single copy gene (S) is represented as T/S ratio which is calculated to determine the relative telomere length. The expression of single copy gene (38B4) was validated using another positive control beta-globin gene. All the samples were run in triplicates in order to minimize the sample to sample variation. The same sample was also repeated in another day and run in triplicates. The p values were the same in different experiements for the same sample or individual. The error value indicates the degree of well to well variation in the 96 well plate used for the PCR experiment. The standard error between the replicate were approximately ≤0.05 (range: 0.02 to 0.05) in most of the samples.

The telomere and single copy gene specific primers used for the experiment were as given below.

Tel forward primer: 5′GGTTTTTGAGGGTGAGGGTGAGGGTGAGGGTGAGGGT 3′

Tel reverse primer: 5′TCCCGACTATCCCTATCCCTATCCCTATCCCTATCCCTA 3′

36B4, forward primer: 5′CAGCAAGTGGGAAGGTGTAATCC 3′

36B4, reverse primer: 5′CCCATTCTATCATCAACGGGTACAA **3′**

Beta globin-forward primer: 5′GCTTCTGACACAACTGTGTTCACTAGC3′

Beta-globin reverse primer, 5′CACCAACTTCATCCACGTTCACC3′

Briefly, PCR reactions were performed in triplicate in 20 µl reaction volumes (using 25 ng DNA sample per reaction) for all the samples studied. The PCR reactions were performed using telomere and single copy gene primers in the same 96 well plate (LC480 light cycler from Roche diagnostics, GmbH, Germany). The PCR mixture contained 10 pmoles of each of the primers, 100 uM of each dNTPs and 0.3 X SYBR green dye and 0.5 Units of fast taq DNA polymerase (Roche Diagnostics, GmbH, Germany). The PCR thermal conditions for relative telomere length assay using telomeric primers (T) and single copy gene primers (S) consisted of a initial denaturation of 5 minutes at 95°C, followed by a total of 40 cycles at 95°C for 5 seconds, 56°C for 30 seconds, and 72°C for 30 seconds and fluorescence acquisition. Crossing points (Cp) were determined using the Light Cycler 480 software (Roche Diagnostics, GmbH, Germany). A standard curve derived from serially-diluted reference DNA was generated in order to check PCR efficiency between the plates. The average of telomere versus single copy gene (T/S) ratio was calculated which is proportional to telomere length of each individual [19]. For quality control purposes, we have repeated many samples that were separately PCR amplified. Majority of the samples were repeated more than twice and run in different plates in order to achieve the consistency. The variation between the plates was <0.05. All the measurements were performed in a blinded fashion without knowledge of sample information.

### Statistical Analysis

Statistical analysis of the samples was performed using the statistical software Sigma stat 3.5 [53]. The relationship between telomere length and radiation dose, effect of gender and age were determined using least squares linear regression analysis. Odds ratio (OR) and their confidence intervals (CI) were calculated in order to see statistical significance in the mean of short and long telomere length among the adults from various dose groups as compared to control. The mean telomere length was determined after adjusting the influence of age and gender, if any, using analysis of Co-Variance (ANCOVA). Using analysis of variance (ANOVA), pairwise comparisons were performed in order to see the differences in the mean telomere length between the groups (normal, and three dose groups from HLNRA). Regression analysis was performed to find out the correlation between telomere length and age. ANOVA/ANCOVA, pairwise comparisons and regression analysis were performed using the software Statosoft [54].
